# The cost effectiveness of intensity-modulated radiation therapy and three-dimensional conformal radiotherapy in the treatment of head and neck cancers

**DOI:** 10.1186/s13014-023-02327-z

**Published:** 2023-08-22

**Authors:** Mehdi Varmaghani, Malihe Amiri, Hossein Ebrahimpour, Roham Salek, Javad Javan-Noughabi

**Affiliations:** 1 Social Determinants of Health Research Center, Mashhad University of Medical Sciences, Mashhad, Iran; 2 Department of Health Economics and Management Sciences, School of Health, Mashhad University of Medical Sciences, Daneshgah st. between 16-18, Mashhad, Iran; 3 Radiotherapy and Oncology Department, Mashhad University of Medical Science, Mashhad, Iran

**Keywords:** Cost-effectiveness analysis, Radiotherapy, Conformal, Radiotherapy, intensity-modulated, Head and Neck Neoplasms

## Abstract

**Purpose:**

Intensity-modulated radiotherapy is developed as a replacement for 3-dimensional conformal radiation therapy. Considering the difference in costs and effectiveness of these interventions, the aim of this study was to compare the cost effectiveness of intensity-modulated radiation therapy and three-dimensional conformal radiotherapy in the treatment of head and neck cancer in east of Iran.

**Methods:**

A Markov model including six states based on xerostomia and dysphagia was developed to estimate the incremental cost effectiveness ratio from the perspective of societal. Cost and quality of life data were collected from 97 respondents via a checklist and EuroQol-5Dimension questionnaire. The robustness of results was examined by deterministic and probabilistic sensitivity analysis. All analysis were conducted with Treeage software.

**Results:**

The results of this study showed that the cost and quality adjusted life years for 3-dimensional conformal radiation therapy were 9209.76 and 3.63 respectively. However, the cost and quality adjusted life years for intensity-modulated radiotherapy were 12562.90 and 3.17 respectively. Therefore, 3-dimensional conformal radiation therapy produced 0.45 more quality adjusted life years than intensity-modulated radiotherapy and saved $3353. According to the incremental cost effectiveness ratio, 3-dimensional conformal radiation therapy as compared to intensity-modulated radiotherapy saved $7367.27 per quality adjusted life years. These results confirmed by sensitivity analysis.

**Conclusion:**

This study concluded that in the treatment of head and neck cancer, the 3-dimensional conformal radiation therapy method appears to be cost-effective when compared with intensity-modulated radiotherapy.

## Introduction

Malignancies arising from a variety of sites including scalp and neck skin, nasal cavity, paranasal sinuses, oral cavity, salivary glands, pharynx and larynx are known as head and neck cancers (HNC) [1, 2]. HNCs are a major cause of morbidity and sixth cause of mortality by cancer [1].

Every year in the world, more than 600,000 new cases of HNC are diagnosed and about one million deaths occur due to HNC [2]. The incidence and prevalence of HNC is different over the world, however, it is most prevalent in Asia and northern Europe [1]. In Iran, due to the lack of a comprehensive cancer registration system, there are no accurate statistics on the incidence and prevalence of HNC. But according to the report of the Ministry of Health, HNC were among the 10 most common cancers in many provinces of the Iran [3].

Given the proximity of head and neck cancers (HNC) to the spinal cord, brain tissue, parathyroid glands, visual system (eye, optic nerve, chiasma), tear glands, and cochlea, they can be not only a serious health threat but also cause a major decline in the patient’s quality of life [4]. There are a variety of treatments for HNC, which attempt to improve the patient’s life expectancy and reduce the risk of recurrence [5].

One common method for treating HNC is radiotherapy, which has undergone significant changes with technological advancements of recent decades, like the replacement of conventional two-dimensional radiotherapy with Three-Dimensional Conformal Radiotherapy (3D-CRT) [6]. With the advancements in treatment planning systems and linear accelerators, many physicians now prefer to use Intensity-Modulated Radiation Therapy (IMRT) instead of 3D-CRT. IMRT adjusts the intensity of radiation to deliver higher doses to the tumor while doing less damage to surrounding healthy tissues [7, 8]. The most important technological advantage of IMRT (over 3D-CRT) for HNC patients is the ability to avoid delivering high radiation doses to parotid glands and pharyngeal constrictor muscles, which reduces the severity of dry mouth and swallowing problems, thereby improving the patient’s quality of life [6, 9]. Intensity-modulated radiation therapy, however, requires more time for treatment planning and physics quality assurance, software upgrades for treatment planning computers, hardware upgrades for linear accelerators allowing modulation of the radiation beam, and an increase in treatment delivery time [6].

However, research has shown that it is far more expensive for HNC patients to undergo IMRT than 3D-CRT. Therefore, while IMRT may provide a better quality of life, it certainly imposes a greater financial burden on patients. A study conducted in the United States has shown that undergoing IMRT instead of conventional radiotherapy will be roughly 6000 dollars more expensive for American HNC patients [10]. According to a study in India, it costs Indian HNC patients 2.3 times more to undergo IMRT than 3D-CRT [11]. Considering the resource limitations of national healthcare systems and the ever-rising healthcare costs, it is important to have a realistic economic assessment of the value of new technologies versus their costs [12–14]. Since there is still not enough evidence of the cost-effectiveness of treating HNC with IMRT or 3D-CRT in Iran, this study conducted an economic evaluation of the use of IMRT versus 3D-CRT to treat HNC in eastern Iran.

## Methods

An economic evaluation was conducted in 2021 to investigate the cost-effectiveness of treating HNC patients with IMRT versus 3D-RCT in east of Iran. The study was carried out in a radiotherapy and oncology clinic that was the only place providing IMRT in the east of Iran. Using the census method, all HNC patients who underwent IMRT or 3DRCT in the clinic in 2021 were included in the study.

**Fig. 1:**
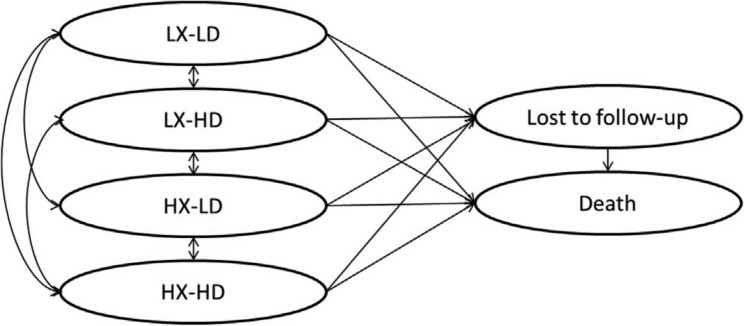
Markov model for IMRT vs. 3D-CRT in the treatment of head and neck cancers

The study was conducted using the Markov model illustrated in Fig. 1, which was set up based on the research performed by Kohler et al. [9].

As shown in the model, the common side effects of radiotherapy for HNC were assumed to be dry mouth and swallowing problems. Patients were divided into two groups in terms of the severity of the disease: mild (stage 0–1) and severe (stage 2 and higher) [15, 16]. According to this, four states were defined as the potential implication of each radiotherapy method: low xerostomia (LX), high xerostomia (HX), low dysphagia (LD), and high dysphagia (HD). Other states were lost to follow up and death. The effects of the two treatment methods were measured using a form prepared in consultation with experts by examining patients’ files. Data for lost to follow-up state were extracted from a trial data. Patients in the lost to follow-up state were assumed to progress to death at the same rate as other cohort members [4, 17].

Cost analysis was conducted by estimating the direct medical costs, direct non-medical costs, and indirect costs of each treatment method using the bottom-up method. Also, we used a human capital approach for calculating the indirect costs. The data needed for this analysis was collected with a checklist created based on previous studies in the field [9, 12, 13].

The checklist consisted of four sections. The first section was devoted to demographic information. The second and third sections were dedicated to the data on the direct medical costs (visits, laboratory, diagnosis and radiotherapy) and direct non-medical costs (transportation and lodging), respectively. The last section was devoted to the estimation of indirect costs, including productivity loss due to patients (and their companions) needing to attend the hospital or patients needing home nursing. These indirect costs were estimated using the human capital approach. Direct medical costs were determined by reviewing patient records and other costs were determined through direct interviews with patients. All costs were converted to US dollars using the average exchange rate Central Bank of Iran for the time period of this study. According to this, one dollar is equal to 36,692 Iranian Rials.

The quality-of-life data was collected with the questionnaire EQ-5D, which measures different aspects of health-related quality of life. In this questionnaire, quality of life is measured in five dimensions of mobility, self-care, usual activities, pain/discomfort, and anxiety/depression, each with three response levels of severity: 1-no problems, 2-some problems, and 3-extreme problems. The 5-digit codes obtained from this questionnaire were transformed into quality-of-life scores based on the tables developed by Goudarzi using the time trade-off (TTO) method for Iran [18]. Transition probabilities were derived from published literature [9].

Finally, data were analyzed by using Treeage software to calculate the cost-effectiveness of the IMRT versus 3D-CRT to treat HNC. To illustrate uncertainty in the results, we performed one way sensitivity analysis, Monte Carlo simulation and drew an acceptability curve to evaluate how the ICERs were influenced by assumptions.

## Results

The total cost imposed by IMRT and 3D-CRT was estimated to be $13,761 and $10,150 (per person) respectively. For both treatment methods, the greatest cost item was the direct medical cost. The total direct medical cost was estimated to be $13,363 for IMRT versus $9772 for 3D-CRT. IMRT also had higher direct non-medical costs than 3D-CRT ($103 for IMRT versus $80 for 3D-CRT). The lost productivity cost of absenteeism was roughly the same for both treatments ($294 for IMRT versus $296 for 3D-CRT) (see Table 1).

**Table 1:** The cost items for IMRT vs. 3D-CRT

Variable	IMRT	3D-CRT
Direct medical costs		
visits	44.17	17.57
Laboratory	138.99	83.12
Diagnosis	184.75	175.17
Radiotherapy	12995.65	9497.05
**Total direct medical costs**	**13363.56**	**9772.92**
Direct non-medical costs		
Transport	46.22	24.89
Inhabitancy	57.14	55.83
**Total direct non-medical costs**	**103.36**	**80.72**
Indirect costs		
Productivity loss due to absent from work	294.41	296.57
**Total cost**	**13761.34**	**10150.22**

**Fig. 2:**
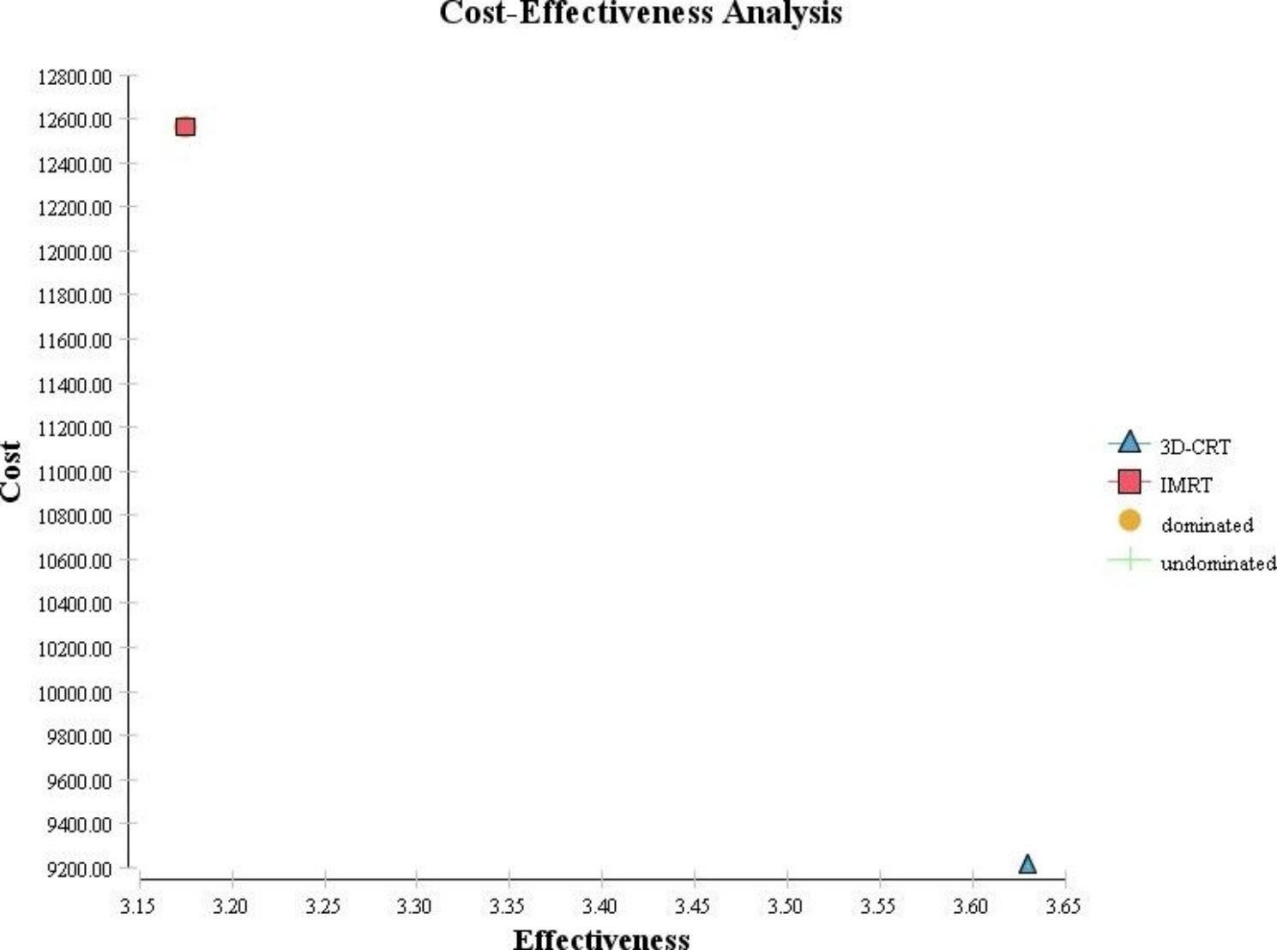
Cost effectiveness analysis of IMRT vs. 3D-CRT in the treatment of head and neck cancers

Table 2 showed the results of cost-effectiveness analysis. Based on these results, it is shown that the incremental cost effectiveness ratio (ICER) is $-7367.27 per QALY. The run of the model shows that 3D-CRT was a cost-effective option for HNC treatment with a cost of $9209.76 and QALY of 3.63. The patients who underwent 3D-CRT had a higher QALY score than those who underwent IMRT (3.63 for 3D-CRT versus 3.17 for IMRT). These findings are also shown graphically in Fig. 2. It is concluded that 3D-CRT was more effective and less costly versus IMRT.

**Table 2:** Cost effectiveness ranking

Strategy	Effectiveness	Incremental effectiveness	Cost	Incremental cost	ICER	Dominance
3D-CRT	3.63	0	9209.76	0	0	
IMRT	3.17	-0.45	12562.90	3353.15	-7367.27	Dominated

**Fig. 3:**
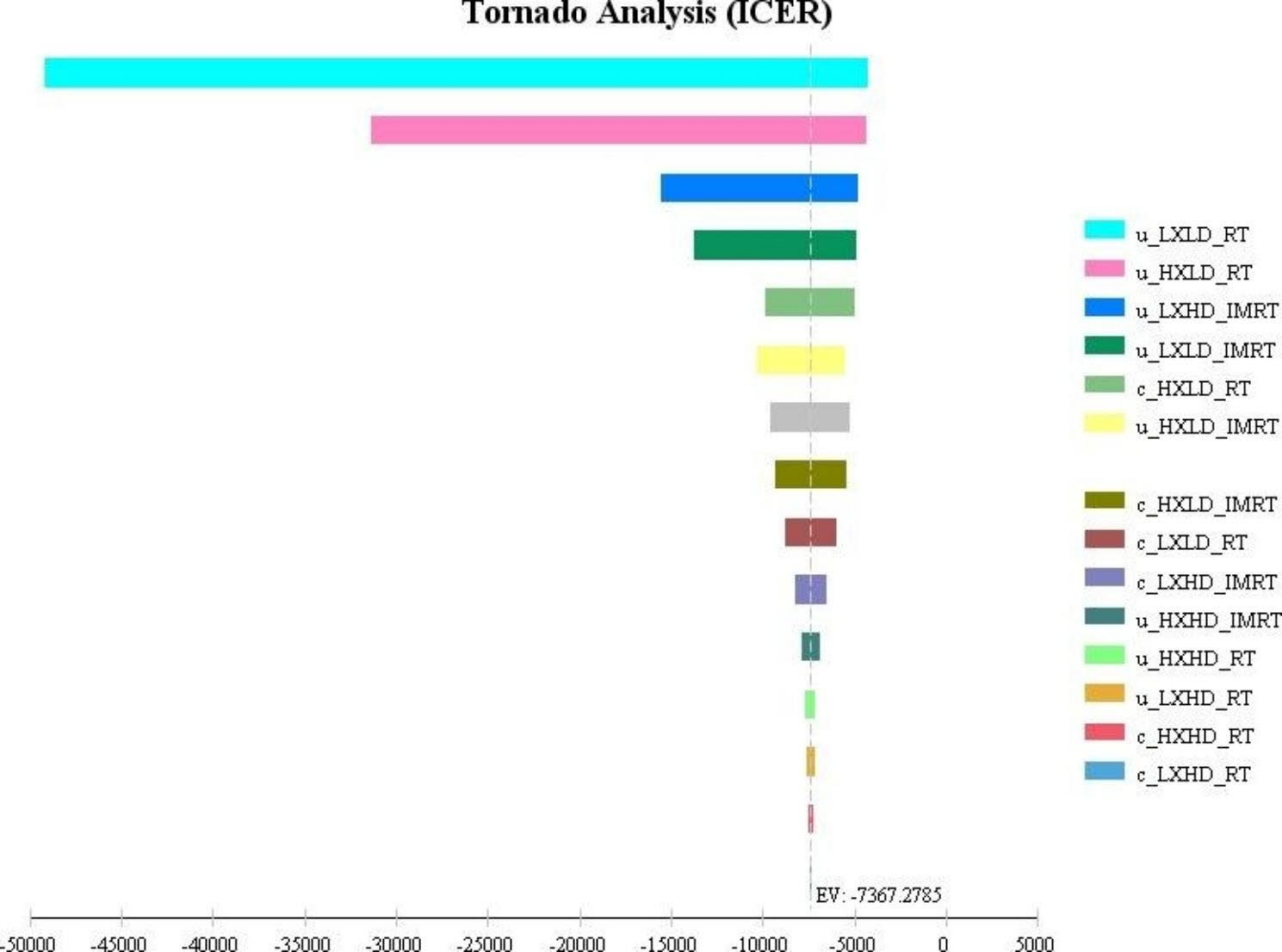
Tornado diagram for multiple one-way sensitivity analysis

These findings are also confirmed by the results of the sensitivity analysis. For this aim, both deterministic and probabilistic sensitivity analysis was carried out to test the robustness of results. The result of the tornado diagram in Fig. 3 indicates that the most influential parameters in the study are the QALY of LXLD and HXLD patients in the 3D-RCT arm.

**Fig. 4:**
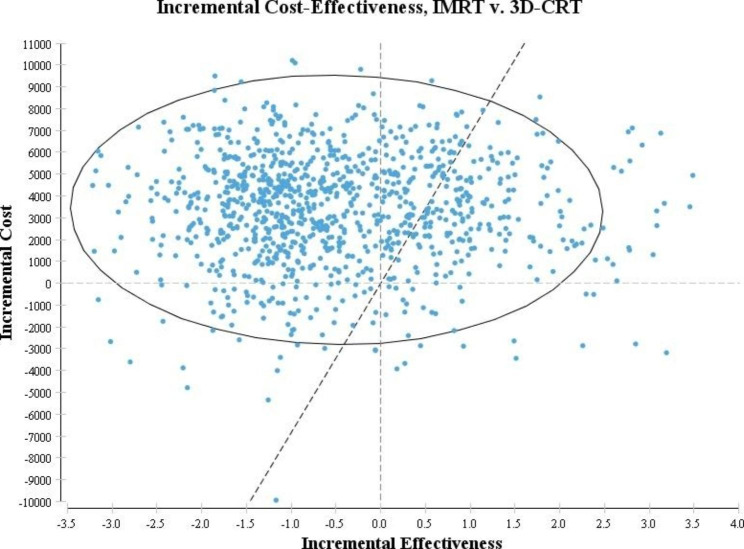
The result of Monte Carlo simulation

**Fig. 5:**
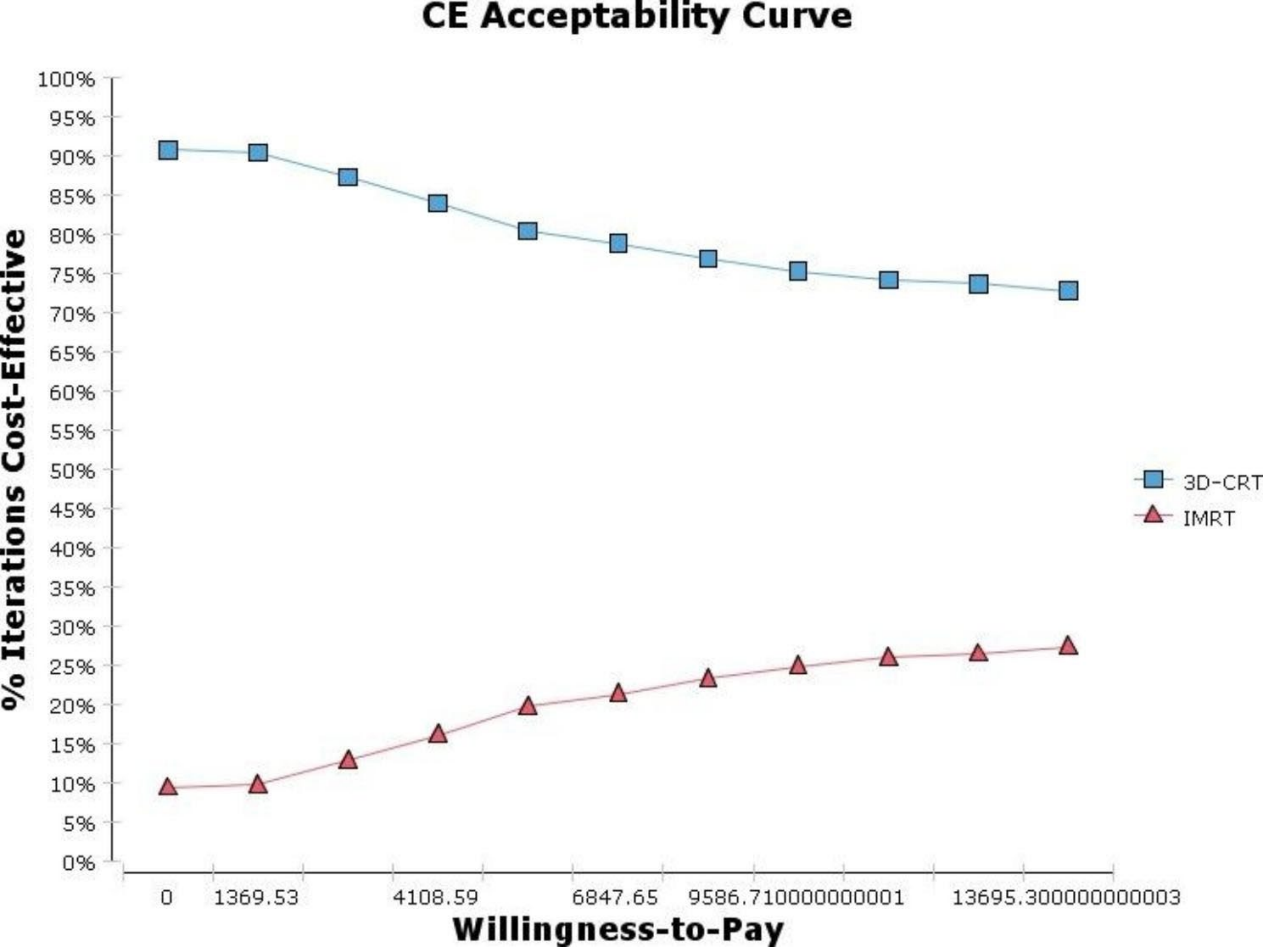
Cost-effectiveness acceptability curve

Probabilistic sensitivity analysis are showed in the Figs. 4 and 5. In Fig. 4, we conducted a Monte Carlo simulation of 1000 hypothetical individuals. The most simulation results placed in the quadrant IV that showed IMRT is less effective and more costly than 3D-CRT. Figure 5 showed the acceptability curves based on the results of Monte Carlo simulation (1000 patients). According this figure, 3D-CRT would be more cost effective at all levels of willingness-to-pay.

## Discussion

Over the years, various radiotherapy techniques have been developed for cancer patients. The latest development in this field is IMRT, which is rapidly becoming the method of choice for treating cancer patients around the world.

However, the findings of our study showed that IMRT imposes a higher cost than 3D-CRT that is consist with other studies. For example, a study by Sheets et al. (2014) that compared the costs of IMRT and 3D-CRT for HNC patients showed that, on average, using IMRT instead of 3D-CRT imposes an extra burden of $5881 on these patients [10]. A study by Kohler et al. (2013) showed that from the perspective of the US healthcare system, IMRT costs over $20,000, which is much higher than the roughly $11,000 cost of 3D-CRT [9]. Marta et al. (2017) also reported that IMRT costs twice as much as 3D-CRT from the perspective of the Brazilian public health system ($10,000 for IMRT versus $5000 for 3D-CRT) [19]. In another study Lester-Coll et al. concluded that the net cost of IMRT was $171,792 net cost of 3DCRT was $163,048 [20]. Chin et al. showed that the median total cost for the IMRT group with $35,890 was higher than the 3D-CRT group with $27,262 [21].

According to the model used and the cost and QALY findings, the calculated ICER in the present study shows that using 3D-CRT instead of IMRT can save $7367. In our study, 3D-CRT cost and effectiveness were $9209 and 3.63, respectively. IMRT cost and effectiveness were $12,562 and 3.17, respectively. Therefore, 3D-CRT was more cost-effective strategy for treating HNC patients.

The cost effectiveness of 3D-CRT versus IMRT in previous studies shows contradictory results.

A study entitled “Cost-Effectiveness of Intensity Modulated Radiation Therapy vs. 3D Conformal Radiation Therapy in Stage III Non-Small Cell Lung Cancer” conducted by Lester-Coll et al. They concluded that the cost-effectiveness of IMRT depends primarily on the WTP threshold. In this study IMRT had more cost but lower QALY than 3D-CRT. costs and QALYs for IMRT were $171,792 and 1.60, respectively. costs and QALYs for 3DCRT were $163,048 and 1.54, respectively [20].

Kohler et al. stated that, with an ICER of $101,100 per QALY gained over a 2-year time horizon, IMRT cannot be considered a cost-effective treatment for HNC in this time horizon; but in a 15-year time horizon, IMRT will have an ICER of $34,523 per QALY gained, which makes it cost-effective [9].

In a study by Chauhan et al. (2020) on the cost-effectiveness of treating HNC with IMRT in India, the findings showed that neither IMRT nor 3D-CRT were cost-effective when compared to 2-DRT. According to this study, in India, IMRT costs $7072 more than 3D-CRT and $5164 more than 2-DRT per patient [22]. However, in Marta et al.’s analysis of the cost-effectiveness of IMRT versus 3D-CRT for Brazilian HNC patients, IMRT was found to be more cost-effective than 3D-CRT over 2 and 15-year time horizons, and their incremental cost-effectiveness ratios (ICER) was estimated to be BRL31579 and BRL4341 per QALY respectively [19]. One of the reasons for the difference in findings can be the use of different costing perspectives and the difference in medical costs in different countries.

Various studies have shown that the use of IMRT is a cost-effective method for prostate cancer patients. Carter et al. in a study in patients post radical prostatectomy from the perspective of the Australian health care system have proved that IMRT was both more effective and less costly than 3DCRT over 20 years [23]. The study of Yong et al. (2012), which was conducted in Canada, also reported that IMRT, with an ICER of $26,768 per QALY gained, was more cost-effective than 3D-CRT in treating prostate cancer [24].

## Conclusion

This study concluded that in the treatment of head and neck cancer, the 3-dimensional conformal radiation therapy method appears to be cost-effective when compared with intensity-modulated radiotherapy.
